# The RUNX Family, a Novel Multifaceted Guardian of the Genome

**DOI:** 10.3390/cells12020255

**Published:** 2023-01-07

**Authors:** Bibek Dutta, Motomi Osato

**Affiliations:** 1 Department of Paediatrics, Yong Loo Lin School of Medicine, National University of Singapore, Singapore 117599, Singapore; 2 Cancer Science Institute of Singapore, National University of Singapore, Singapore 117599, Singapore; 3 International Research Center for Medical Sciences, Kumamoto University, Kumamoto 860-0811, Japan

**Keywords:** RUNX, DNA repair, retrotransposon, telomere, p53

## Abstract

The DNA repair machinery exists to protect cells from daily genetic insults by orchestrating multiple intrinsic and extrinsic factors. One such factor recently identified is the Runt-related transcription factor (RUNX) family, a group of proteins that act as a master transcriptional regulator for multiple biological functions such as embryonic development, stem cell behaviors, and oncogenesis. A significant number of studies in the past decades have delineated the involvement of RUNX proteins in DNA repair. Alterations in RUNX genes cause organ failure and predisposition to cancers, as seen in patients carrying mutations in the other well-established DNA repair genes. Herein, we review the currently existing findings and provide new insights into transcriptional and non-transcriptional multifaceted regulation of DNA repair by RUNX family proteins.

## 1. Introduction

Human cells are continuously exposed to endogenous and exogenous deadly insults that can result in severely adverse conditions such as cancer and premature aging. Cells encounter frequent endogenous damage from reactive oxygen species (ROS) and replication errors. The replication of a single human cell requires high-fidelity copying of 3 × 10^9^ bases by DNA polymerases [1]. This complicated process is not completely error-proof, and as a result, base substitutions and insertions/deletions (indels) accumulate at a rate of 10^−6^ to 10^−8^ per generation of the cell cycle [2].

Fortunately, there exists an exemplary cellular machinery that can recognize and repair diverse DNA lesions (Table 1), thereby maintaining genomic integrity. This complex evolutionarily conserved cellular mechanism is called the DNA repair machinery. The repair machinery comprises multiple different mechanisms to cope with DNA damage. This DNA repair machinery is not flawless. Sometimes the damage is beyond repair, and the cell is fated to either enter senescence or die.

This DNA repair machinery works through cohesive interaction among a multitude of cellular factors. The discoveries of such DNA repair factors were frequently initiated by their unique link to radiation-related episodes. RUNX family genes also have a tight relationship with radiation. A study of radiation-related myelodysplastic syndrome (MDS) and acute myeloid leukemia (AML) patients among Hiroshima atomic bomb survivors showed that 46% of the patients who succumbed to the disease carried point mutations in the *RUNX1* gene [3]. Moreover, US soldiers and residents who were exposed to radiation from the aboveground nuclear bomb tests in Bikini atoll or Nevada from the 1940s to 1950s developed MDS/AML harboring RUNX1 point mutations or RUNX1-related chromosomal translocation [4]. Secondly, it is well documented that RUNX1 point mutations were observed in 38% of the patients who developed secondary MDS/AML after successful treatment against primary cancers with chemotherapeutic agents with or without radiation therapy [3].

Besides the unique radiation-related phenomenon, the association with lymphocyte development, where DNA recombination is required, also suggests the potential involvement of a factor in DNA repair machinery. RUNX family proteins are well known to be involved with VDJ recombination [5,6,7]. VDJ recombination is essential for B and T lymphocyte development to be fully functional in the immune system [8,9]. VDJ recombination involves double-strand break (DSB) formation by RAG recombinases and repair through non-homologous end joining (NHEJ), and any defect in this process leads to diseases associated with compromised immunity in humans [9]. *Runx1* conditional knockout (KO) mice exhibited lymphocyte development defects due to abrogated VDJ recombination [10,11,12,13]. Moreover, direct and indirect interaction of the RUNX family with RAG and NHEJ genes has been shown [12,13,14,15,16,17].

The frequent involvement of RUNX family genes in lymphocyte development and diseases associated with radiation and chemotherapy suggests the potential roles of RUNX proteins in DNA damage repair.

## 2. RUNX Manages Reactive Oxygen Species (ROS) and Oxidative Stress

The mutations in RUNX proteins observed in patients exposed to radiation or chemotherapeutic drugs imply the importance of RUNX proteins in the downstream DNA repair pathways that deal with exogenous insults. Apart from exogenous agents, DNA damage also occurs from exposure to endogenous agents. Most of the endogenous damage arises from the interaction of DNA with reactive oxygen species (ROS) and the integration of retrotransposons [endogenous retrovirus (ERV), long interspersed class 1 elements (LINE-1)] [18,19]. RUNX proteins have been shown to directly regulate the endogenous levels of ROS and retrotransposable elements (RTEs).

**Figure 1:**
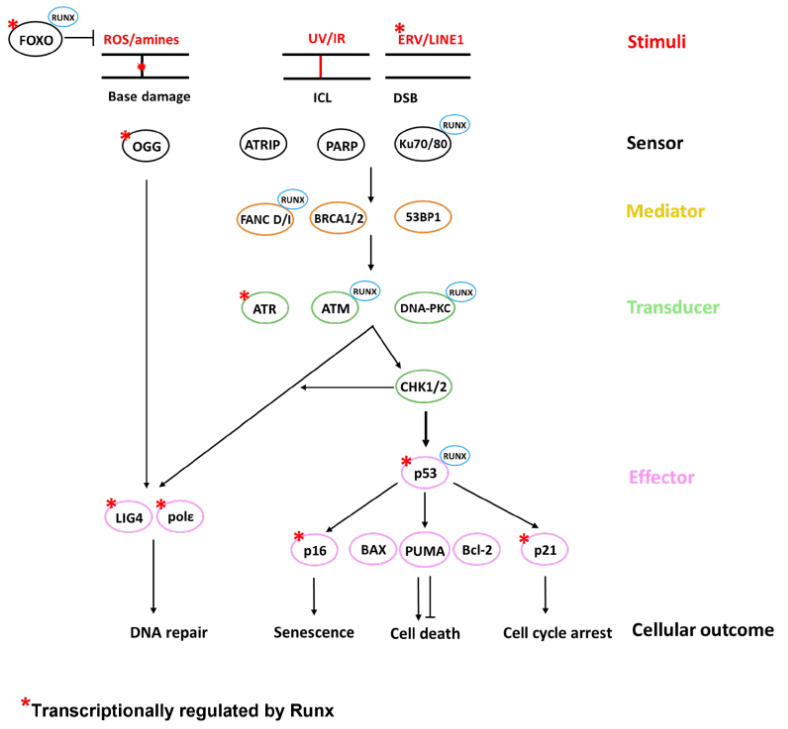
Involvement of the RUNX family in DNA repair pathways. In the scenario where a cell encounters DNA damage, the cell decides on the specific DNA repair pathway responsible for that damage. The DNA repair process is broadly divided into 5 stages: stimulus response, damage sensor, signal transducer, and mediators that provide binding of different factors and effectors. Based on the extent of the damage, the DNA might be repaired or, otherwise, the cell is fated toward apoptosis or senescence. RUNX proteins are involved in every one of these stages, from the regulation of endogenous damaging agents such as ROS and endogenous retrovirus elements (ERV/LINE-1) to the mediation of cell fate by interacting with p53 and its downstream genes. RUNX proteins regulate the DNA repair machinery either by interacting directly with the participating proteins or through transcriptional regulation. DNA repair factors that are transcriptionally regulated by RUNX1 are marked with a red asterisk. Abbreviations: ROS, reactive oxygen species; UV, ultraviolet; IR, ionizing radiation; ERV, endogenous retrovirus; ICL, inter-strand cross link; DSB, double-strand break.

ROS are endogenous agents that are by-products of the electron transport chain (ETC) [20]. These ROS form base lesions and strand breaks by damaging the methyl group and sugar residues [21,22,23]. RUNX proteins are involved in the maintenance of redox balance. ROS accumulation was hindered in leukemia-initiating cells (LIC) in T-cell acute lymphoblastic leukemia (T-ALL) through the downregulation of the protein kinase C θ (*PKC θ*) gene by NOTCH1 in a RUNX-dependent manner [24,25]. RUNX3 induced by NOTCH1 represses RUNX1, leading to the induction of PKC θ. This model postulates that an increase in RUNX1 leads to increased oxidative stress and premature senescence, but there exists another model that contradicts the role of RUNX1 in ROS manipulation. Single-cell gene expression profiling of breast acinar morphogenesis showed an inhibitor of ROS, the Forkhead transcription factor *FOXO1*, as a target gene of RUNX1 (Figure 1). This model showed that inhibition of RUNX1 and, subsequently, FOXO1 leads to an increase in overall oxidative stress [26]. Although the two models contradict each other, RUNX1 seems to play an important role in the maintenance of redox balance. Moreover, like RUNX1, RUNX3 was observed to interact with another member of the FOX gene family, FOXO3a [27]. Furthermore, in the non-small cell lung cancer model, ROS produced via ectopic expression of TGFβ was counteracted by RUNX3. RUNX3 elevates the expression of redox regulator *HMOX1*, which catalyzes the production of anti-oxidant bilirubin [28]. The other member of the family, RUNX2, may also play a role in the maintenance of ROS during osteoblastic differentiation. *RUNX2* was among the genes downregulated in hydrogen-peroxide-treated MC3T3-E1 cells [29]. Although further investigation is still needed, these findings suggest that the RUNX family takes part in the reduction of ROS, the largest endogenous genetic insult to the genome.

## 3. RUNX Regulates Retrotransposable Elements (RTEs)

Some viruses serve as exogenous DNA damage agents [30]. Retroviruses directly introduce genomic lesions as they integrate their proviruses into the host mammalian genome [31]. Notably, RUNX proteins were originally discovered as transcription factors (TFs) that bind to viral enhancers present in the regulatory region, particularly in MoMuLV [32]. Although RUNX proteins were shown to be a positive regulator for viral propagation [33,34], RUNX proteins were also shown to play a suppressive role. The unique relationship between RUNX proteins and retrovirus was recently revisited in HIV. RUNX heterodimerization factor CBFβ has been shown to bind with and stabilize the virus infectivity factor (Vif) of HIV which degrades APOBEC3 (A3). This CBFβ–Vif complex negatively impacts the transcription of RUNX-associated genes, some of which are related to DNA repair [35]. Furthermore, over-expression of RUNX1 was reported to reduce the expression of HIV-1 viral proteins and their replication [36].

The eukaryotic genome is heavily occupied (>45%) by a highly repetitive genetic component which shares similarities with retroviruses and is movable in the genome in a copy and paste manner. These elements, termed RTEs, are broadly classified into long terminal repeat (LTR) and non-log terminal repeat (non-LTR) RTEs. The expression of RTEs is involved in multiple diseases, including cancer, and can be used as a prognosis marker in AML [37]. AML with RUNX1 point mutation and inv(16) carrying CBFβ alteration showed increased RTE expression and fell into the high-risk category.

The LTR RTE family includes the ERV subfamily. ERVs constitute almost 8% of the human genome. Most of the ERV elements are non-infectious and have lost the ability to transpose in their genome due to a lack of flanking LTRs [38], but the replication and transposition of ERV can occur with the help of an autonomous retroelement, LINE-1 (L1) [39]. The regulatory regions of the ERVs include binding sites of several TFs such as RUNX1 and ETS [40]. Loss-of-function experiments on the ERV regulatory region pointed out its importance in hematopoietic development and immunity [40,41,42]. Moreover, the deregulation of ERV elements is associated with AML. Chromatin immunoprecipitation assays with sequencing (ChIP-seq) studies in AML cell lines showed clear binding enrichment of RUNX1 in ERV, and deletion of these ERV LTRs led to apoptosis in AML cell lines [43].

The other family, non-LTR RTEs, includes L1 and short interspersed elements (SINE). L1s constitute almost 17% of the human genome. L1 transcription is facilitated through a sense promoter that produces two proteins, ORF1p and ORF2p. ORF1p is involved in nuclear chaperone activity, while ORF2p provides the reverse transcription and endonuclease activity. The expression of L1 RNA and ORF proteins may occur in almost all types of cells, though the levels of expression are usually very low in the majority of somatic cells [44]. The transposability of the L1 elements makes them one of the top endogenous DNA-damaging agents [45]. Interestingly, L1 expression is modulated by the RUNX family (Figure 1). Human L1 promoter consists of RUNX3 binding sites [46,47]. Overexpression of RUNX3 leads to increased expression of L1, while RUNX1 and RUNX2 have suppressive effects [47]. Recent studies showed that irradiation increases L1 expression in hematopoietic cells, and the elevated L1 is lowered through interferon signaling mediated by exogenous thrombopoietin (TPO) [48]. RUNX1 is a transcriptional regulator of thrombopoietin receptor (MPL) and is involved with IFN-γ signaling, both of which regulate the L1 expression levels. Therefore, RUNX proteins appear to play a key role in L1 transcription. L1 insertion also leads to aberrant gene expression. One of the notable target genes of L1 insertion is *RUNX1*. A genomic insertion of L1 increased RUNX1 transcripts by (26 ± 8)-fold in human embryonic stem cells (hESCs) [49]. Considering *RUNX1* involvement in embryonic and hematopoietic development, L1-mediated abnormal *RUNX1* expression may lead to leukemia and embryonic defects. These findings support the possibility of a tight relationship between RUNX protein and L1.

## 4. RUNX Proteins Function in the Central DNA Repair Mechanism

DNA repair is an extremely complicated process. Distinct repair mechanisms are employed against different types of damage (Table 1, Figure 1). ROS- and chemical-reaction-mediated base modifications are repaired by the base excision repair pathway (BER), whereas the nucleotide excision repair pathway (NER) takes care of the nucleotide modifications imparted by UV and chemical components. Indels and base misincorporations occurring during replication errors are repaired by the mismatch repair pathway (MMR). The most dangerous form of DNA lesions, double-strand breaks (DSBs), are repaired either by the faithful homologous recombination pathway (HR) or by the error-prone NHEJ pathway. There exists another form of damage: strand crosslinks which can arise from irradiation and chemotherapeutic agents. The repair of crosslinks employs the activation of the Fanconi anemia (FA), HR, NER, and translesion synthesis (TLS) pathways.

Several cellular factors are involved in the DNA repair pathways. Amongst these proteins, a family of TFs, namely, the RUNX family, is becoming more relevant in the field of DNA repair. The RUNX family comprises RUNX1, RUNX2, and RUNX3. RUNX proteins play essential roles in several biological processes [50,51,52], and knockout of two of these proteins in mice leads to perinatal lethality [51,53,54]. The RUNX family is involved in all layers of the DNA repair machinery (Table 1, Figure 1). It has also been shown that the RUNX family exerts its DNA repair function not only via transcriptional regulation with the help of CBFβ but also through non-transcriptional regulation via physically interacting with other known DNA repair molecules.

RUNX has been shown to regulate the BER pathway. The RUNX1–ETO fusion protein, which is found in t(8:21) leukemia and functions as a dominant negative form against wild-type RUNX1, was shown to cause the downregulation of eight genes involved in BER [55,56]. Moreover, RUNX1–ETO-expressing cells were inefficient in repairing 8-oxoGuanine (8-oxoG), which is repaired by 8-oxoguanine DNA glycosylase (*OGG1*), one of the above-mentioned downregulated BER pathway genes [57,58]. These cells showed heightened sensitivity to PARP inhibitors and significant downregulation of the HR and FA pathways [59]. Further extensive studies on the role of RUNX in the BER pathway remain to be conducted.

RUNX3, a popular tumor suppressor in gastric cancer, was found to be directly associated with the sensor protein complex Ku70/80 of the NHEJ pathway through its transactivation domain (TAD) [16]. However, how this protein complex impacts the NHEJ pathway remains to be elucidated. Apart from the association with the sensor proteins, RUNX3 also directly binds with the transducer protein ATM. RUNX3 was shown to form a complex with a phosphorylated form of ATM in HeLa cells after treatment with adriamycin (ADR). It was further elucidated that RUNX3 acts as a recruiting factor of ATM onto p53 when cells are exposed to DNA damage [60]

In recent studies, RUNX proteins were shown to play a critical role in the ICL repair mediated by the FA pathway. Double knockout (DKO) mice of Runx1 and Runx3 showed co-occurrence of bone marrow failure (BMF) and myeloproliferative disorder (MPD). These phenotypes are seen in patients suffering from FA syndrome, suggesting the potential role of RUNX1 and RUNX3 in FA-pathway-mediated DNA repair [61]. Indeed, the DKO mice showed heightened sensitivity to DNA-damaging agents such as mitomycin C, which is commonly used for FA diagnosis. Mechanistically, it was further observed that both RUNX proteins physically and functionally interact with FANCI and FANCD2. Depletion of RUNX proteins significantly hampers the recruitment of FANC proteins. This recruitment of FANC proteins is independent of the RUNX/CBF-β heterodimerization-mediated transcription, suggesting non-transcriptional control on DNA repair machinery. Subsequent studies on the interaction of RUNX and FANC proteins showed that RUNX1 and RUNX3 are both poly(ADP-ribosyl)ated in a PARP-dependent manner, thereby interacting with Bloom syndrome protein (BLM) when DNA damage is introduced [62]. This interaction modulates the recruitment of FANCI/D2 in the DNA damage foci.

Damage to individual base positions also arises during replication errors, which might result in random indels and misincorporations. Dysregulation of MMR pathway genes have been associated with several cancers [63]. Two of the genes associated with the MMR pathway, *MSH6* and *SETD2,* were among the genes found to be frequently mutated in relapsed or therapy resistant ETV6/RUNX1 acute lymphoblastic leukemia (ALL) [64,65]. Mutations in MMR genes were also associated with RUNX1-mutated blast-phase chronic myeloid leukemia (BP-CML) [66,67]. Furthermore, isolated myeloid sarcoma patients (IMS) with germline *MSH6* mutation often carried a mutation in the *RUNX1* gene [68]. Apart from RUNX1, RUNX3 also seems to be involved in the MMR pathway. Knockout mouse models for *Mlh3* and *Pms2* displayed increased gastrointestinal tumor progression. Further investigation revealed Tle6-like or *TLE6D,* a member of the Transducin enhancer of Split (Tle) family, as the tumor-related amplified gene. TLE6D directly interacts with *RUNX3* and mediates the inhibition of RUNX3 transcription [69]. These studies suggest the possible involvement of RUNX proteins in MMR regulation.

## 5. RUNX Proteins May Maintain Telomere Length

Telomeres are repetitive DNA sequences of TTAGGG that form a cap and protect all the chromosomal ends [70,71]. These cap-like structures are extremely crucial for genome integrity, and telomeric dysfunction leads to devastating conditions. In the studies about RUNX proteins in FA pathways, DKO of RUNX1 and RUNX3 led to increased radiosensitivity [60], a phenotype that can result from telomere shortening [72]. Moreover, FA patients and mice deficient in the DNA repair proteins Atm, Parp, DNA-Pkcs, and Ku were shown to have altered telomere maintenance [73,74,75]. These correlations suggest a potential connection between telomere maintenance and DNA repair.

Extensive studies in yeast, SCID mice, and mammalian cells revealed the role of Ku proteins in the maintenance of telomeres [76,77,78,79]. Ku does not bind directly to the telomere DNA, but it facilitates the localization of telomeric repeat binding factors (TRF), TRF1 and TRF2, in the telomeric repeats [80,81]. Another protein of the NHEJ pathway that plays a role in telomere maintenance is DNA-PKC [82]. Inactivation of DNA-PKCs in mouse embryonic fibroblasts (MEFs) causes telomere fusions, suggesting the importance of DNA-PKCs in capping the chromosome ends. Other DNA repair proteins related to telomere maintenance are PARP-1 and ATM [73,83].

Proteins Ku, DNA-PKCs, and PARP-1 have been shown to directly interact with RUNX3. Hela-S3 cells expressing FLAG-tagged RUNX3 were subjected to SILAC (stable isotope labeling by amino acids in cell culture) to identify the RUNX3 interacting proteins. Apart from the DNA repair proteins involved in telomere maintenance, proteins involved in the cap complex and telomere maturation complex were also identified [62].

Additionally, the correlation between telomere length (TL) and mutation profile of 30 myeloid genes from 67 AML patients showed that TL is regulated by RUNX1. Patients with FLT3 and RUNX1 mutations, t(8;21), or inv(16) showed a trend towards short TL with a p-value of 0.026 [84]. Moreover, a comparison of TLs amongst patients carrying mutations in the genes related to bone marrow failure demonstrated that patients with mutations in LIG4 or RUNX1 had the shortest TLs [85]. MDS patients with RUNX1 mutations also showed a trend towards shorter TL, though it was not statistically significant [86].

Apart from its association with TL, RUNX1 has also been associated with the expression of *TERT*, one of the components of telomerase. The RUNX1-null human embryonic stem cell line GIBHe008 displayed a reduction in TERT [87]. Furthermore, RUNX1 has been observed to modulate the expression of genes such as *SIRT1* and CEBPα, which have been shown to directly regulate the expression of *TERT* [88,89,90,91]. Interestingly, recent studies have shown that TERT and TERC play an integral role in regulating the expression of RUNX2 [92,93]. These results suggest that RUNX proteins and telomere maintenance genes share an integral relationship, though further studies will be required to evaluate their in-depth relationship.

## 6. RUNX Modulates p53-Dependent Cell Death

In the scenario where DNA repair fails, one of the cell fates is apoptosis, which occurs through p53-dependent and -independent manners. RUNX family proteins are involved in modulating p53 activity either by direct interaction or through transcriptional regulation.

All three of these RUNX proteins physically interact with p53 after the induction of DNA damage by ADR, but they differ in their ways of influencing the p53 activity [60,94,95]. RUNX1 acts as a scaffold for p53–p300 binding, which facilitates the acetylation of p53 at Lys-373/382 residues [94], while RUNX3 mediates ADR-induced phosphorylation of p53 at the Ser-15 residue [60]. Unlike with RUNX1 and RUNX3, both the phosphorylation and acetylation of p53 are inhibited in the presence of RUNX2. The deacetylation of p53 was later found to be mediated by HDAC6, which acts as a binding partner of RUNX2 [95]. Apart from its role in the modification of p53, the RUNX/p53 complex was also found to transcriptionally regulate the expression of p53 downstream genes. The expression of pro-apoptotic genes BAX, NOXA, and PUMA shared a direct correlation with RUNX1 and RUNX3 expression and a negative correlation with RUNX2 expression (Figure 1).

**Figure 2:**
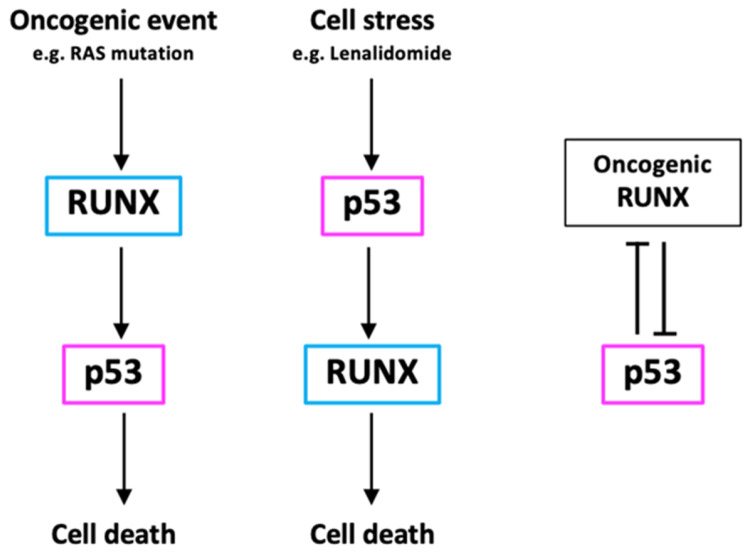
Bidirectional relationship between RUNX and p53. Wild-type RUNX and p53 positively regulate each other via transcriptional and non-transcriptional mechanisms dependent on the type of stimulation, whereas oncogenic RUNX and wild-type p53 negatively regulate one another.

An association between RUNX proteins and p53 has also been documented at steady state without NDA damage-inducing stress. Hematopoietic stem cells (HSCs) from *Runx1*-deficient mice displayed lower p53 protein levels but did not show a reduction in total mRNA levels, indicating the involvement of RUNX1 in the post-translational modification of p53 [96]. Similarly to Runx1 protein deficiency, loss of Runx1 methylation in HSCs also resulted in the abrogation of p53-dependent transcription and attenuation of apoptosis [97]. The transcriptional activity of RUNX1 is affected by the methylation loss, suggesting that RUNX1 also transcriptionally modulates p53. Furthermore, ChIP-seq data of RUNX1 displayed RUNX1 peaks at the p53 promoter region (data not shown). RUNX3 has also been shown to regulate p53 activity in the presence of oncogenic *RAS* expression [98]. Oncogenic RAS leads to RUNX3 activation via the MAPK pathway, and RUNX3, in turn, forms a complex with BRD2. The RUNX3/BRD2 complex induces ARF, which stabilizes p53 through the suppression of MDM2 [98]. These studies further support the notion that RUNX proteins modulate p53 through direct physical interaction and transcriptional regulation (Figure 2).

The relationship between p53 and RUNX proteins is not one-directional. p53 has also been shown to regulate RUNX levels. Lenalidomide resistance in MDS cells is conferred through mutations in or downregulation of RUNX1. Both RUNX1 mRNA and protein levels were observed to be significantly reduced in p53 KO MDS cells after lenalidomide treatment, suggesting that the downregulation of RUNX1 is induced by p53 deficiency [99]. Although RUNX proteins are primarily considered tumor suppressors, multiple studies have also shown that RUNX can also act as potential oncogenes and that p53 suppresses RUNX expression. RUNX1 inhibition often coincides with the upregulation of p53 and CBFβ in some leukemic cells. RUNX1-p53-CBFβ forms a feedback regulatory loop. RUNX1 inhibition in AML cells leads to p53 induction, which results in higher expression levels of CBFβ [100]. The elevated CBFβ stabilizes the depleted RUNX1 levels, conferring therapy resistance. Moreover, CBFβ expression was also upregulated in p53-deficient osteosarcoma, where CBFβ formed a stable complex with RUNX2 [101]. These studies suggest that p53 transcriptionally regulates RUNX proteins through CBFβ. Similar to RUNX1, RUNX3 and p53 form a regulatory axis through MDM2 expression. The induction of p53 stimulates MDM2 production in cells, which, in turn, ubiquitinates key lysine residues of RUNX3, causing proteasomal degradation [102]. Furthermore, RUNX protein levels along with MYC expression were found to be consistently upregulated in p53-deficient cancers [101,103,104]. These results suggest that p53 acts as a negative regulator for RUNX and MYC expression (Figure 2). 

## 7. RUNX Proteins Induce Senescence

Apart from cell death, failure of DNA repair leads to senescence. All three RUNX proteins have been shown to induce senescence in primary MEFs in a p53-dependent manner [59,105]. Ectopic expression of RUNX in the presence of an oncogene induces senescence via the upregulation of p19^ARF^.

RUNX1 and RUNX1–ETO both have been shown to induce senescence in primary fibroblasts and hematopoietic progenitors [59,106,107,108,109]. RUNX1 displayed senescence induction through elevated levels of p19^ARF^ expression in primary MEFs [59,109]. Similar to RUNX1, RUNX1–ETO-mediated senescence induction is dependent on p53 but independent of p19^ARF^/p14^ARF^ and p16^INK4a^ [107,109]. RUNX1–ETO is a potent inducer of ROS, activating the p38^MAPK^ pathway and elevating p53 protein levels, leading to senescence [107]. Although RUNX1–ETO expression leads to senescence, it also induces potent senescence-associated secretory phenotype (SASP), which ultimately results in escape from senescence [108]. RUNX3 acts as a regulator for ARF and p21 expression during the activation of oncogenic Ras [98]. Activation of K-RAS triggers the formation of the RUNX3–BRD2 complex, which induces the expression of ARF and p21 [98]. RUNX3 dissociates from BRD2 upon deactivation of K-RAS and forms a complex with HDAC4, which deacetylates RUNX3 and suppresses ARF and p21 expression [110]. Moreover, RUNX3 has also been reported to be a critical factor in inhibiting the progression of hepatocellular carcinoma via senescence [111]. Although RUNX2 negatively regulates the apoptotic activity of p53, it was shown that RUNX2-null MEFs escaped H-Ras^V12^-mediated senescence. This escape from senescence happened even in the presence of upregulated p38MAPK, p53, p21^Waf1^, p16^Ink4a^, and p19^Arf^. This observation suggests a role of RUNX2 downstream of the Ras/p38MAPK/p53 pathway. This phenotype was the result of elevated expression of S/G2/M cyclin genes caused by defective E2F: pRb: SWI/SNF-dependent gene repression [105,112]. These results suggest that RUNX2 may be an integral part of the SWI/SNF complex. Further studies may provide a deeper understanding of the role of RUNX proteins in senescence.

## 8. Therapeutic Applications

Mutation in the RUNX family genes causes a predisposition to cancer [113,114,115,116], at least in part due to a defective DNA repair system [117,118,119,120]. Therefore, therapeutic agents against cells carrying defective DNA pathways, such as PARP inhibitors, can be used to sensitize the cancer cells to traditional cancer therapies (chemotherapy and radiation therapy) [121,122,123,124]. Indeed, RUNX1–ETO-expressing AML cell lines were shown to be sensitive to PARP inhibitors [125,126,127]. The tight relation between *p53* gene activity and the RUNX family also opens the possibility to utilize MDM2 inhibitors [128,129,130]. MDM2, a ubiquitin ligase, functions as the principal cellular antagonist of p53 [131]. Inhibition of MDM2 will help to stabilize the impaired p53 activity in RUNX1/3-deficient cells, thus promoting apoptosis in malignant cells.

## 9. Conclusions

The evidence so far depicts the involvement of the RUNX family at multiple levels within the DNA repair process, from the regulation of stimuli to cellular outcome. Notably, RTE suppression and telomere maintenance are summarized in this review for the first time as previously unappreciated DNA-repair-related pathways mediated by RUNX. RUNX proteins perform this multi-layered regulation either through direct physical interaction or via the transcriptional regulation of genes involved with DNA repair machinery or their downstream genes. The review only summarizes the initial observations about the roles of RUNX family proteins as a new guardian of the genome. Several important questions such as those surrounding RUNX involvement in NER remain to be addressed. Further investigations need to be made to deepen our understanding about the roles of the RUNX family in DNA repair.

## Figures and Tables

**Table 1:** Involvement of the RUNX family in DNA repair pathways.

Stimuli	DNA Damage Lesions	Repair Mechanism	RUNX Involvement
ROS Hydrolysis Alkylating agents Aromatic amines	Abasic sites SSBs 8-oxo-G	BER	Yes
UV Chemical agents	DNA adducts Pyrimidine dimers Glycols DNA–protein crosslink	NER	No?
IR Chemotherapeutic drugs	SSBs DSBs ICL DNA–protein crosslink	HR, FA	Yes
		NHEJ	Yes
Replication stress	Indels Base mismatch	MMR	Yes?
	Telomere erosion	TERT, TERC, ALT	Yes?
ERV, LINE-1	DSBs Indels Integration Retrotransduction	HR NHEJ MMR MMEJ	Yes?

Abbreviations: ROS, reactive oxygen species; SSB, single strand break; BER, base excision repair; NER, nucleotide excision repair; UV, ultraviolet; IR, ionizing radiation; DSB, double-strand break; ICL, inter-strand cross link; HR, homologous recombination; FA, Fanconi anemia; NHEJ, non-homologous recombination; Indel, insertion/deletion; MMR, mismatch repair; ALT, alternative lengthening of telomeres; ERV, endogenous retrovirus; MMEJ, microhomology-mediated end joining.

## Data Availability

Not applicable.

## References

[1] Loeb L.A., Monnat R.J. (2008). DNA polymerases and human disease. Nat. Rev. Genet..

[2] Kunkel T.A. (2004). DNA replication fidelity. J. Biol. Chem..

[3] Harada H., Harada Y., Tanaka H., Kimura A., Inaba T. (2003). Implications of somatic mutations in the AML1 gene in radiation-associated and therapy-related myelodysplastic syndrome/acute myeloid leukemia. Blood.

[4] Osato M. (2004). Point mutations in the RUNX1/AML1 gene: Another actor in RUNX leukemia. Oncogene.

[5] Mevel R., Draper J.E., Lie-a-Ling M., Kouskoff V., Lacaud G. (2019). RUNX transcription factors: Orchestrators of development. Development.

[6] Seo W., Muroi S., Akiyama K., Taniuchi I. (2017). Distinct requirement of Runx complexes for TCRβ enhancer activation at distinct developmental stages. Sci. Rep..

[7] Proudhon C., Hao B., Raviram R., Chaumeil J., Skok J.A. (2015). Long-Range Regulation of V(D)J Recombination. Adv. Immunol..

[8] Dudley D.D., Sekiguchi J., Zhu C., Sadofsky M.J., Whitlow S., DeVido J., Monroe R.J., Bassing C.H., Alt F.W. (2003). Impaired V(D)J recombination and lymphocyte development in core RAG1-expressing mice. J. Exp. Med..

[9] Jung D., Alt F.W. (2004). Unraveling V(D)J Recombination: Insights into Gene Regulation. Cell.

[10] Egawa T., Tillman R.E., Naoe Y., Taniuchi I., Littman D.R. (2007). The role of the Runx transcription factors in thymocyte differentiation and in homeostasis of naive T cells. J. Exp. Med..

[11] Seitz V., Kleo K., Dröge A., Schaper S., Elezkurtaj S., Bedjaoui N., Dimitrova L., Sommerfeld A., Berg E., von der Wall E. (2020). Evidence for a role of RUNX1 as recombinase cofactor for TCRβ rearrangements and pathological deletions in ETV6-RUNX1 ALL. Sci. Rep..

[12] Cieslak A., Le Noir S., Trinquand A., Lhermitte L., Franchini D.-M., Villarese P., Gon S., Bond J., Simonin M., Vanhille L. (2014). RUNX1-dependent RAG1 deposition instigates human TCR-δ locus rearrangement. J. Exp. Med..

[13] Seo W., Ikawa T., Kawamoto H., Taniuchi I. (2012). Runx1–Cbfβ facilitates early B lymphocyte development by regulating expression of Ebf1. J. Exp. Med..

[14] Papaemmanuil E., Rapado I., Li Y., Potter N.E., Wedge D.C., Tubio J., Alexandrov L.B., Van Loo P., Cooke S.L., Marshall J. (2014). RAG-mediated recombination is the predominant driver of oncogenic rearrangement in ETV6-RUNX1 acute lymphoblastic leukemia. Nat. Genet..

[15] Jakobczyk H., Jiang Y., Debaize L., Soubise B., Avner S., Sérandour A.A., Rouger-Gaudichon J., Rio A.-G., Carroll J.S., Raslova H. (2022). ETV6-RUNX1 and RUNX1 directly regulate RAG1 expression: One more step in the understanding of childhood B-cell acute lymphoblastic leukemia leukemogenesis. Leukemia.

[16] Tanaka Y., Imamura J., Kanai F., Ichimura T., Isobe T., Koike M., Kudo Y., Tateishi K., Ikenoue T., Ijichi H. (2007). Runx3 interacts with DNA repair protein Ku70. Exp. Cell Res..

[17] Willis D.M., Loewy A.P., Charlton-Kachigian N., Shao J.S., Ornitz D.M., Towler D.A. (2002). Regulation of osteocalcin gene expression by a novel Ku antigen transcription factor complex. J. Biol. Chem..

[18] Farkash E.A., Luning Prak E.T. (2006). DNA damage and L1 retrotransposition. J. Biomed. Biotechnol..

[19] Maxwell P.H., Burhans W.C., Curcio M.J. (2011). Retrotransposition is associated with genome instability during chronological aging. Proc. Natl. Acad. Sci..

[20] Dahlgren C., Karlsson A. (1999). Respiratory burst in human neutrophils. J. Immunol. Methods.

[21] Hall E.J., Rossi H.H., Kellerer A.M., Goodman L., Marino S. (1973). Radiobiological Studies with Monoenergetic Neutrons. Radiat. Res..

[22] Cooke M.S., Evans M.D., Dizdaroglu M., Lunec J. (2003). Oxidative DNA damage: Mechanisms, mutation, and disease. FASEB J..

[23] Salehi F., Behboudi H., Kavoosi G., Ardestani S.K. (2018). Oxidative DNA damage induced by ROS-modulating agents with the ability to target DNA: A comparison of the biological characteristics of citrus pectin and apple pectin. Sci. Rep..

[24] Giambra V., Jenkins C.E., Lam S.H., Hoofd C., Belmonte M., Wang X., Gusscott S., Gracias D., Weng A.P. (2015). Leukemia stem cells in T-ALL require active Hif1α and Wnt signaling. Blood.

[25] Giambra V., Jenkins C.R., Wang H., Lam S.H., Shevchuk O.O., Nemirovsky O., Wai C., Gusscott S., Chiang M.Y., Aster J.C. (2012). NOTCH1 promotes T cell leukemia-initiating activity by RUNX-mediated regulation of PKC-θ and reactive oxygen species. Nat. Med..

[26] Wang L., Brugge J.S., Janes K.A. (2011). Intersection of FOXO- and RUNX1-mediated gene expression programs in single breast epithelial cells during morphogenesis and tumor progression. Proc. Natl. Acad. Sci. USA.

[27] Yamamura Y., Lee W.L., Inoue K., Ida H., Ito Y. (2006). RUNX3 cooperates with FoxO3a to induce apoptosis in gastric cancer cells. J. Biol. Chem..

[28] Krishnan V., Chong Y.L., Tan T.Z., Kulkarni M., Bin Rahmat M.B., Tay L.S., Sankar H., Jokhun D.S., Ganesan A., Chuang L.S.H. (2018). TGFβ Promotes Genomic Instability after Loss of RUNX3. Cancer Res..

[29] Arai M., Shibata Y., Pugdee K., Abiko Y., Ogata Y. (2007). Effects of reactive oxygen species (ROS) on antioxidant system and osteoblastic differentiation in MC3T3-E1 cells. IUBMB Life.

[30] Weitzman M.D., Lilley C.E., Chaurushiya M.S. (2010). Genomes in Conflict: Maintaining Genome Integrity during Virus Infection. Annu. Rev. Microbiol..

[31] Skalka A.M., Katz R.A. (2005). Retroviral DNA integration and the DNA damage response. Cell Death Differ..

[32] Ito Y. (2008). RUNX genes in development and cancer: Regulation of viral gene expression and the discovery of RUNX family genes. Adv. Cancer Res..

[33] Huan C., Li Z., Ning S., Wang H., Yu X.F., Zhang W. (2018). Long Noncoding RNA uc002yug.2 Activates HIV-1 Latency through Regulation of mRNA Levels of Various RUNX1 Isoforms and Increased Tat Expression. J. Virol..

[34] Hu Y., Pan Q., Zhou K., Ling Y., Wang H., Li Y. (2022). RUNX1 inhibits the antiviral immune response against influenza A virus through attenuating type I interferon signaling. Virol. J..

[35] Kim D.Y., Kwon E., Hartley P.D., Crosby D.C., Mann S., Krogan N.J., Gross J.D. (2013). CBFβ stabilizes HIV Vif to counteract APOBEC3 at the expense of RUNX1 target gene expression. Mol. Cell.

[36] Klase Z., Yedavalli V.S.R.K., Houzet L., Perkins M., Maldarelli F., Brenchley J., Strebel K., Liu P., Jeang K.-T. (2014). Activation of HIV-1 from Latent Infection via Synergy of RUNX1 Inhibitor Ro5-3335 and SAHA. PLoS Pathog..

[37] Colombo A., Triche T., Ramsingh G. (2017). Transposable Element Expression Predicts Prognosis in Acute Myeloid Leukemia. bioRxiv.

[38] Thomas J., Perron H., Feschotte C. (2018). Variation in proviral content among human genomes mediated by LTR recombination. Mob. DNA.

[39] Naveira H., Bello X., Abal-Fabeiro J.L., Maside X. (2014). Evidence for the persistence of an active endogenous retrovirus (ERVE) in humans. Genetica.

[40] Ye M., Goudot C., Hoyler T., Lemoine B., Amigorena S., Zueva E. (2020). Specific subfamilies of transposable elements contribute to different domains of T lymphocyte enhancers. Proc. Natl. Acad. Sci. USA.

[41] Pi W., Zhu X., Wu M., Wang Y., Fulzele S., Eroglu A., Ling J., Tuan D. (2010). Long-range function of an intergenic retrotransposon. Proc. Natl. Acad. Sci. USA.

[42] Chuong E.B., Elde N.C., Feschotte C. (2016). Regulatory evolution of innate immunity through co-option of endogenous retroviruses. Science.

[43] Deniz Ö., Ahmed M., Todd C.D., Rio-Machin A., Dawson M.A., Branco M.R. (2020). Endogenous retroviruses are a source of enhancers with oncogenic potential in acute myeloid leukaemia. Nat. Commun..

[44] Macia A., Widmann T.J., Heras S.R., Ayllon V., Sanchez L., Benkaddour-Boumzaouad M., Muñoz-Lopez M., Rubio A., Amador-Cubero S., Blanco-Jimenez E. (2017). Engineered LINE-1 retrotransposition in nondividing human neurons. Genome Res..

[45] Zhang X., Zhang R., Yu J. (2020). New Understanding of the Relevant Role of LINE-1 Retrotransposition in Human Disease and Immune Modulation. Front. Cell Dev. Biol..

[46] Lee S.-H., Cho S.-Y., Shannon M.F., Fan J., Rangasamy D. (2010). The Impact of CpG Island on Defining Transcriptional Activation of the Mouse L1 Retrotransposable Elements. PLoS ONE.

[47] Yang N., Zhang L., Zhang Y., Kazazian H.H. (2003). An important role for RUNX3 in human L1 transcription and retrotransposition. Nucleic Acids Res..

[48] Barbieri D., Elvira-Matelot E., Pelinski Y., Genève L., de Laval B., Yogarajah G., Pecquet C., Constantinescu S.N., Porteu F. (2018). Thrombopoietin protects hematopoietic stem cells from retrotransposon-mediated damage by promoting an antiviral response. J. Exp. Med..

[49] Muñoz-Lopez M., Vilar R., Philippe C., Rahbari R., Richardson S.R., Andres-Anton M., Widmann T., Cano D., Cortes J.L., Rubio-Roldan A. (2019). LINE-1 retrotransposition impacts the genome of human pre-implantation embryos and extraembryonic tissues. bioRxiv.

[50] Okuda T., van Deursen J., Hiebert S.W., Grosveld G., Downing J.R. (1996). AML1, the target of multiple chromosomal translocations in human leukemia, is essential for normal fetal liver hematopoiesis. Cell.

[51] Komori T., Yagi H., Nomura S., Yamaguchi A., Sasaki K., Deguchi K., Shimizu Y., Bronson R.T., Gao Y.H., Inada M. (1997). Targeted disruption of Cbfa1 results in a complete lack of bone formation owing to maturational arrest of osteoblasts. Cell.

[52] Li Q.L., Ito K., Sakakura C., Fukamachi H., Inoue K., Chi X.Z., Lee K.Y., Nomura S., Lee C.W., Han S.B. (2002). Causal relationship between the loss of RUNX3 expression and gastric cancer. Cell.

[53] Wang Q., Stacy T., Binder M., Marin-Padilla M., Sharpe A.H., Speck N.A. (1996). Disruption of the Cbfa2 gene causes necrosis and hemorrhaging in the central nervous system and blocks definitive hematopoiesis. Proc. Natl. Acad. Sci. USA.

[54] Levanon D., Groner Y. (2009). Runx3-deficient mouse strains circa 2008: Resemblance and dissimilarity. Blood Cells Mol. Dis..

[55] Alcalay M., Meani N., Gelmetti V., Fantozzi A., Fagioli M., Orleth A., Riganelli D., Sebastiani C., Cappelli E., Casciari C. (2003). Acute myeloid leukemia fusion proteins deregulate genes involved in stem cell maintenance and DNA repair. J. Clin. Investig..

[56] Krejci O., Wunderlich M., Geiger H., Chou F.S., Schleimer D., Jansen M., Andreassen P.R., Mulloy J.C. (2008). p53 signaling in response to increased DNA damage sensitizes AML1-ETO cells to stress-induced death. Blood.

[57] Liddiard K., Hills R., Burnett A.K., Darley R.L., Tonks A. (2010). OGG1 is a novel prognostic indicator in acute myeloid leukaemia. Oncogene.

[58] Forster V.J., Nahari M.H., Martinez-Soria N., Bradburn A.K., Ptasinska A., Assi S.A., Fordham S.E., McNeil H., Bonifer C., Heidenreich O. (2016). The leukemia-associated RUNX1/ETO oncoprotein confers a mutator phenotype. Leukemia.

[59] Wotton S.F., Blyth K., Kilbey A., Jenkins A., Terry A., Bernardin-Fried F., Friedman A.D., Baxter E.W., Neil J.C., Cameron E.R. (2004). RUNX1 transformation of primary embryonic fibroblasts is revealed in the absence of p53. Oncogene.

[60] Wang C.Q., Krishnan V., Tay L.S., Chin D.W., Koh C.P., Chooi J.Y., Nah G.S., Du L., Jacob B., Yamashita N. (2014). Disruption of Runx1 and Runx3 leads to bone marrow failure and leukemia predisposition due to transcriptional and DNA repair defects. Cell Rep..

[61] Tay L.S., Krishnan V., Sankar H., Chong Y.L., Chuang L.S.H., Tan T.Z., Kolinjivadi A.M., Kappei D., Ito Y. (2018). RUNX Poly(ADP-Ribosyl)ation and BLM Interaction Facilitate the Fanconi Anemia Pathway of DNA Repair. Cell Rep..

[62] Pećina-Šlaus N., Kafka A., Salamon I., Bukovac A. (2020). Mismatch Repair Pathway, Genome Stability and Cancer. Front. Mol. Biosci..

[63] Kuster L., Grausenburger R., Fuka G., Kaindl U., Krapf G., Inthal A., Mann G., Kauer M., Rainer J., Kofler R. (2011). ETV6/RUNX1-positive relapses evolve from an ancestral clone and frequently acquire deletions of genes implicated in glucocorticoid signaling. Blood.

[64] Mar B.G., Bullinger L.B., McLean K.M., Grauman P.V., Harris M.H., Stevenson K., Neuberg D.S., Sinha A.U., Sallan S.E., Silverman L.B. (2014). Mutations in epigenetic regulators including SETD2 are gained during relapse in paediatric acute lymphoblastic leukaemia. Nat. Commun..

[65] Adnan Awad S., Dufva O., Ianevski A., Ghimire B., Koski J., Maliniemi P., Thomson D., Schreiber A., Heckman C.A., Koskenvesa P. (2021). RUNX1 mutations in blast-phase chronic myeloid leukemia associate with distinct phenotypes, transcriptional profiles, and drug responses. Leukemia.

[66] Awad S., Kankainen M., Dufva O., Heckman C.A., Porkka K., Mustjoki S. (2018). RUNX1 Mutations Identify an Entity of Blast Phase Chronic Myeloid Leukemia (BP-CML) Patients with Distinct Phenotype, Transcriptional Profile and Drug Vulnerabilities. Blood.

[67] Liu Y., GuLiBaHa M., Yue Y.B., Li M.W., Cao S.B., Yan M. (2021). An isolated childhood myeloid sarcoma with germline MSH6 mutation-a case report. Transl. Pediatr..

[68] Chen P.C., Kuraguchi M., Velasquez J., Wang Y., Yang K., Edwards R., Gillen D., Edelmann W., Kucherlapati R., Lipkin S.M. (2008). Novel roles for MLH3 deficiency and TLE6-like amplification in DNA mismatch repair-deficient gastrointestinal tumorigenesis and progression. PLoS Genet..

[69] Blackburn E.H. (1991). Structure and function of telomeres. Nature.

[70] Counter C.M., Avilion A.A., LeFeuvre C.E., Stewart N.G., Greider C.W., Harley C.B., Bacchetti S. (1992). Telomere shortening associated with chromosome instability is arrested in immortal cells which express telomerase activity. EMBO J..

[71] Slijepcevic P. (2004). Is there a link between telomere maintenance and radiosensitivity?. Radiat. Res..

[72] Hande M.P., Balajee A.S., Tchirkov A., Wynshaw-Boris A., Lansdorp P.M. (2001). Extra-chromosomal telomeric DNA in cells from Atm(−/−) mice and patients with ataxia-telangiectasia. Hum. Mol. Genet..

[73] Callén E., Samper E., Ramírez M.J., Creus A., Marcos R., Ortega J.J., Olivé T., Badell I., Blasco M.A., Surrallés J. (2002). Breaks at telomeres and TRF2-independent end fusions in Fanconi anemia. Hum. Mol. Genet..

[74] Goytisolo F.A., Blasco M.A. (2002). Many ways to telomere dysfunction: In vivo studies using mouse models. Oncogene.

[75] Baumann P., Cech T.R. (2000). Protection of telomeres by the Ku protein in fission yeast. Mol. Biol. Cell.

[76] Boulton S.J., Jackson S.P. (1998). Components of the Ku-dependent non-homologous end-joining pathway are involved in telomeric length maintenance and telomeric silencing. EMBO J..

[77] Hande P., Slijepcevic P., Silver A., Bouffler S., van Buul P., Bryant P., Lansdorp P. (1999). Elongated telomeres in scid mice. Genomics.

[78] d’Adda di Fagagna F., Hande M.P., Tong W.M., Roth D., Lansdorp P.M., Wang Z.Q., Jackson S.P. (2001). Effects of DNA nonhomologous end-joining factors on telomere length and chromosomal stability in mammalian cells. Curr. Biol..

[79] Hsu H.L., Gilley D., Galande S.A., Hande M.P., Allen B., Kim S.H., Li G.C., Campisi J., Kohwi-Shigematsu T., Chen D.J. (2000). Ku acts in a unique way at the mammalian telomere to prevent end joining. Genes Dev..

[80] Ribes-Zamora A., Indiviglio S.M., Mihalek I., Williams C.L., Bertuch A.A. (2013). TRF2 interaction with Ku heterotetramerization interface gives insight into c-NHEJ prevention at human telomeres. Cell Rep..

[81] Gilley D., Tanaka H., Hande M.P., Kurimasa A., Li G.C., Oshimura M., Chen D.J. (2001). DNA-PKcs is critical for telomere capping. Proc. Natl. Acad. Sci. USA.

[82] d’Adda di Fagagna F., Hande M.P., Tong W.M., Lansdorp P.M., Wang Z.Q., Jackson S.P. (1999). Functions of poly(ADP-ribose) polymerase in controlling telomere length and chromosomal stability. Nat. Genet..

[83] Watts J.M., Dumitriu B., Hilden P., Chen C., Rapaport F., Kishtagari A., Ahn J., Devlin S.M., Rampal R.K., Levine R.L. (2014). Telomere Length Is Associated with Specific Mutations and Mutation Classes in Patients with Acute Myeloid Leukemia. Blood.

[84] Alder J.K., Hanumanthu V.S., Strong M.A., DeZern A.E., Stanley S.E., Takemoto C.M., Danilova L., Applegate C.D., Bolton S.G., Mohr D.W. (2018). Diagnostic utility of telomere length testing in a hospital-based setting. Proc. Natl. Acad. Sci. USA.

[85] Hwang S.M., Kim S.Y., Kim J.A., Park H.S., Park S.N., Im K., Kim K., Kim S.M., Lee D.S. (2016). Short telomere length and its correlation with gene mutations in myelodysplastic syndrome. J. Hematol. Oncol..

[86] Ullah H., You H., Shah Z., Fan C., Zhang B., Liu H., Zhang J., Abbas N., Filonenko E.S., Samokhvalov I.M. (2020). Generation of RUNX1-null reporter human embryonic stem cell line GIBHe008-A. Stem Cell Res..

[87] Huang P., Riordan S.M., Heruth D.P., Grigoryev D.N., Zhang L.Q., Ye S.Q. (2015). A critical role of nicotinamide phosphoribosyltransferase in human telomerase reverse transcriptase induction by resveratrol in aortic smooth muscle cells. Oncotarget.

[88] Zhou L., Wang Q., Chen X., Fu L., Zhang X., Wang L., Deng A., Li D., Liu J., Lv N. (2017). AML1–ETO promotes SIRT1 expression to enhance leukemogenesis of t(8;21) acute myeloid leukemia. Exp. Hematol..

[89] Bertran L., Eigbefoh-Addeh A., Portillo-Carrasquer M., Barrientos-Riosalido A., Binetti J., Aguilar C., Ugarte Chicote J., Bartra H., Artigas L., Coma M. (2022). Identification of the Potential Molecular Mechanisms Linking RUNX1 Activity with Nonalcoholic Fatty Liver Disease, by Means of Systems Biology. Biomedicines.

[90] Kumar M., Witt B., Knippschild U., Koch S., Meena J.K., Heinlein C., Weise J.M., Krepulat F., Kuchenbauer F., Iben S. (2013). CEBP factors regulate telomerase reverse transcriptase promoter activity in whey acidic protein-T mice during mammary carcinogenesis. Int. J. Cancer.

[91] Gao G.C., Yang D.W., Liu W. (2020). LncRNA TERC alleviates the progression of osteoporosis by absorbing miRNA-217 to upregulate RUNX2. Eur. Rev. Med. Pharmacol. Sci..

[92] Cuevas R.A., Hortells L., Chu C.C., Wong R., Crane A., Boufford C., Regan C., Moorhead W.J., Bashline M.J., Parwal A. (2022). Non-canonical Telomerase Reverse Transcriptase Controls Osteogenic Differentiation of Aortic Valve Cells Through STAT5. bioRxiv.

[93] Wu D., Ozaki T., Yoshihara Y., Kubo N., Nakagawara A. (2013). Runt-related transcription factor 1 (RUNX1) stimulates tumor suppressor p53 protein in response to DNA damage through complex formation and acetylation. J. Biol. Chem..

[94] Yamada C., Ozaki T., Ando K., Suenaga Y., Inoue K., Ito Y., Okoshi R., Kageyama H., Kimura H., Miyazaki M. (2010). RUNX3 modulates DNA damage-mediated phosphorylation of tumor suppressor p53 at Ser-15 and acts as a co-activator for p53. J. Biol. Chem..

[95] Ozaki T., Wu D., Sugimoto H., Nagase H., Nakagawara A. (2013). Runt-related transcription factor 2 (RUNX2) inhibits p53-dependent apoptosis through the collaboration with HDAC6 in response to DNA damage. Cell Death Dis..

[96] Cai X., Gao L., Teng L., Ge J., Oo Z.M., Kumar A.R., Gilliland D.G., Mason P.J., Tan K., Speck N.A. (2015). Runx1 Deficiency Decreases Ribosome Biogenesis and Confers Stress Resistance to Hematopoietic Stem and Progenitor Cells. Cell Stem Cell.

[97] Matsumura T., Nakamura-Ishizu A., Muddineni S., Tan D.Q., Wang C.Q., Tokunaga K., Tirado-Magallanes R., Sian S., Benoukraf T., Okuda T. (2020). Hematopoietic stem cells acquire survival advantage by loss of RUNX1 methylation identified in familial leukemia. Blood.

[98] Lee Y.S., Lee J.W., Jang J.W., Chi X.Z., Kim J.H., Li Y.H., Kim M.K., Kim D.M., Choi B.S., Kim E.G. (2013). Runx3 inactivation is a crucial early event in the development of lung adenocarcinoma. Cancer Cell.

[99] Martinez-Høyer S., Deng Y., Parker J., Jiang J., Mo A., Docking T.R., Gharaee N., Li J., Umlandt P., Fuller M. (2020). Loss of lenalidomide-induced megakaryocytic differentiation leads to therapy resistance in del(5q) myelodysplastic syndrome. Nat. Cell Biol..

[100] Morita K., Noura M., Tokushige C., Maeda S., Kiyose H., Kashiwazaki G., Taniguchi J., Bando T., Yoshida K., Ozaki T. (2017). Autonomous feedback loop of RUNX1-p53-CBFB in acute myeloid leukemia cells. Sci. Rep..

[101] Shin M.H., He Y., Marrogi E., Piperdi S., Ren L., Khanna C., Gorlick R., Liu C., Huang J. (2016). A RUNX2-Mediated Epigenetic Regulation of the Survival of p53 Defective Cancer Cells. PLoS Genet..

[102] Chi X.Z., Kim J., Lee Y.H., Lee J.W., Lee K.S., Wee H., Kim W.J., Park W.Y., Oh B.C., Stein G.S. (2009). Runt-related transcription factor RUNX3 is a target of MDM2-mediated ubiquitination. Cancer Res..

[103] Date Y., Taniuchi I., Ito K. (2022). Oncogenic Runx1-Myc axis in p53-deficient thymic lymphoma. Gene.

[104] Otani S., Date Y., Ueno T., Ito T., Kajikawa S., Omori K., Taniuchi I., Umeda M., Komori T., Toguchida J. (2022). Runx3 is required for oncogenic Myc upregulation in p53-deficient osteosarcoma. Oncogene.

[105] Kilbey A., Blyth K., Wotton S., Terry A., Jenkins A., Bell M., Hanlon L., Cameron E.R., Neil J.C. (2007). Runx2 disruption promotes immortalization and confers resistance to oncogene-induced senescence in primary murine fibroblasts. Cancer Res..

[106] Wajapeyee N., Wang S.Z., Serra R.W., Solomon P.D., Nagarajan A., Zhu X., Green M.R. (2010). Senescence induction in human fibroblasts and hematopoietic progenitors by leukemogenic fusion proteins. Blood.

[107] Wolyniec K., Wotton S., Kilbey A., Jenkins A., Terry A., Peters G., Stocking C., Cameron E., Neil J.C. (2009). RUNX1 and its fusion oncoprotein derivative, RUNX1-ETO, induce senescence-like growth arrest independently of replicative stress. Oncogene.

[108] Anderson G., Mackay N., Gilroy K., Hay J., Borland G., McDonald A., Bell M., Hassanudin S.A., Cameron E., Neil J.C. (2018). RUNX-mediated growth arrest and senescence are attenuated by diverse mechanisms in cells expressing RUNX1 fusion oncoproteins. J. Cell Biochem..

[109] Linggi B., Müller-Tidow C., van de Locht L., Hu M., Nip J., Serve H., Berdel W.E., van der Reijden B., Quelle D.E., Rowley J.D. (2002). The t(8;21) fusion protein, AML1–ETO, specifically represses the transcription of the p14ARF tumor suppressor in acute myeloid leukemia. Nat. Med..

[110] Jin Y.H., Jeon E.J., Li Q.L., Lee Y.H., Choi J.K., Kim W.J., Lee K.Y., Bae S.C. (2004). Transforming growth factor-beta stimulates p300-dependent RUNX3 acetylation, which inhibits ubiquitination-mediated degradation. J. Biol. Chem..

[111] Chen Z., Zuo X., Pu L., Zhang Y., Han G., Zhang L., Wu J., Wang X. (2019). circLARP4 induces cellular senescence through regulating miR-761/RUNX3/p53/p21 signaling in hepatocellular carcinoma. Cancer Sci..

[112] Bulavin D.V., Kovalsky O., Hollander M.C., Fornace A.J. (2003). Loss of oncogenic H-ras-induced cell cycle arrest and p38 mitogen-activated protein kinase activation by disruption of Gadd45a. Mol. Cell Biol..

[113] Ito Y., Bae S.-C., Chuang L.S.H. (2015). The RUNX family: Developmental regulators in cancer. Nat. Rev. Cancer.

[114] Osato M., Yanagida M., Shigesada K., Itoa Y. (2001). Point Mutations of the RUNX1/AML1 Gene in Sporadic and Familial Myeloid Leukemias. Int. J. Hematol..

[115] Otálora-Otálora B.A., Henríquez B., López-Kleine L., Rojas A. (2019). RUNX family: Oncogenes or tumor suppressors (Review). Oncol. Rep..

[116] Chuang L.S.H., Ito K., Ito Y. (2013). RUNX family: Regulation and diversification of roles through interacting proteins. Int. J. Cancer.

[117] Jackson S.P., Bartek J. (2009). The DNA-damage response in human biology and disease. Nature.

[118] Halazonetis T.D., Gorgoulis V.G., Bartek J. (2008). An oncogene-induced DNA damage model for cancer development. Science.

[119] Ashworth A. (2008). A synthetic lethal therapeutic approach: Poly(ADP) ribose polymerase inhibitors for the treatment of cancers deficient in DNA double-strand break repair. J. Clin. Oncol..

[120] Curtin N.J. (2012). DNA repair dysregulation from cancer driver to therapeutic target. Nat. Rev. Cancer.

[121] Helleday T. (2011). The underlying mechanism for the PARP and BRCA synthetic lethality: Clearing up the misunderstandings. Mol. Oncol..

[122] Murai J., Huang S.Y., Das B.B., Renaud A., Zhang Y., Doroshow J.H., Ji J., Takeda S., Pommier Y. (2012). Trapping of PARP1 and PARP2 by Clinical PARP Inhibitors. Cancer Res..

[123] Lu Y., Liu Y., Pang Y., Pacak K., Yang C. (2018). Double-barreled gun: Combination of PARP inhibitor with conventional chemotherapy. Pharmacol. Ther..

[124] Barcellini A., Loap P., Murata K., Villa R., Kirova Y., Okonogi N., Orlandi E. (2021). PARP Inhibitors in Combination with Radiotherapy: To Do or Not to Do?. Cancers.

[125] Li X., Li C., Jin J., Wang J., Huang J., Ma Z., Huang X., He X., Zhou Y., Xu Y. (2018). High PARP-1 expression predicts poor survival in acute myeloid leukemia and PARP-1 inhibitor and SAHA-bendamustine hybrid inhibitor combination treatment synergistically enhances anti-tumor effects. EBioMedicine.

[126] Li D., Luo Y., Chen X., Zhang L., Wang T., Zhuang Y., Fan Y., Xu J., Chen Y., Wu L. (2019). NF-κB and Poly (ADP-ribose) Polymerase 1 Form a Positive Feedback Loop that Regulates DNA Repair in Acute Myeloid Leukemia Cells. Mol. Cancer Res..

[127] Nieborowska-Skorska M., Paietta E.M., Levine R.L., Fernandez H.F., Tallman M.S., Litzow M.R., Skorski T. (2019). Inhibition of the mutated c-KIT kinase in AML1-ETO-positive leukemia cells restores sensitivity to PARP inhibitor. Blood Adv..

[128] Vazquez A., Bond E.E., Levine A.J., Bond G.L. (2008). The genetics of the p53 pathway, apoptosis and cancer therapy. Nat. Rev. Drug Discov..

[129] Issaeva N., Bozko P., Enge M., Protopopova M., Verhoef L.G., Masucci M., Pramanik A., Selivanova G. (2004). Small molecule RITA binds to p53, blocks p53-HDM-2 interaction and activates p53 function in tumors. Nat. Med..

[130] Kaindl U., Morak M., Portsmouth C., Mecklenbräuker A., Kauer M., Zeginigg M., Attarbaschi A., Haas O.A., Panzer-Grümayer R. (2014). Blocking ETV6/RUNX1-induced MDM2 overexpression by Nutlin-3 reactivates p53 signaling in childhood leukemia. Leukemia.

[131] Iwakuma T., Lozano G. (2003). MDM2, an introduction. Mol. Cancer Res..

